# Why pulsatility still matters: a review of current knowledge

**DOI:** 10.3325/cmj.2014.55.609

**Published:** 2014-12

**Authors:** Davor Barić

**Affiliations:** Department of Cardiac Surgery and Transplantation, Dubrava University Hospital, Zagreb, Croatia

## Abstract

Continuous-flow left ventricular assist devices (LVAD) have become standard therapy option for patients with advanced heart failure. They offer several advantages over previously used pulsatile-flow LVADs, including improved durability, less surgical trauma, higher energy efficiency, and lower thrombogenicity. These benefits translate into better survival, lower frequency of adverse events, improved quality of life, and higher functional capacity of patients. However, mounting evidence shows unanticipated consequences of continuous-flow support, such as acquired aortic valve insufficiency and acquired von Willebrand syndrome. In this review article we discuss current evidence on differences between continuous and pulsatile mechanical circulatory support, with a focus on clinical implications and potential benefits of pulsatile flow.

During the last few decades, mechanical circulatory support has evolved into a standard therapy for patients with advanced heart failure – as a bridge to cardiac transplantation (1-3), bridge to myocardial recovery (4-7), or as destination therapy (8-10). This success can in most part be attributed to the use of continuous-flow devices and their advantages over previously used pulsatile pumps: they offer improved durability, less surgical trauma due to their smaller size, higher energy efficiency, and lower thrombogenicity. These benefits translate into better survival, lower frequency of adverse events, improved quality of life, and higher functional capacity of patients (11-13).

The debate on the importance of pulsatility began several decades ago with the research on the effects of nonpulsatile flow during the cardiopulmonary bypass (CPB) (14,15). It is still alive today, after nearly a decade of use of continuous-flow devices, especially after evidence has shown that this therapy is complicated by diminished pulsatility. In this review article we discuss current evidence on differences between continuous and pulsatile ventricular assist devices, with a focus on clinical implications and potential benefits of pulsatile flow.

## Methods

We searched MEDLINE (via PubMed), EMBASE, and Cochrane Library databases. Search terms included, but were not limited to the medical subject headings (MeSH terms) or key words, such as “pulsatile flow,” “continuous flow,” or “ventricular assist device.” The articles were selected based on the titles and abstracts, and full-text articles were acquired. Additionally, reference lists of selected articles were explored for additional related studies. The literature review finally included 97 studies.

## PULSATILITY

Pulsatility is one of intrinsic properties of the cardiovascular system and has been the focus of extensive research for ages, ever since the time of Aristotle and Avicenna. However, it was not until much later that the mechanism of pulse generation was investigated and understood.

The basic principle of pulse generation is as follows: when the ventricle contracts and creates the needed pressure gradient, a volume of blood is rapidly ejected into the arterial vessels. The aorta and arteries have a lower resistance to blood flow compared to the arterioles and capillaries. Due to the slower outflow to the arteriole, the arteries are inflated to accommodate the extra blood volume. During diastole, the elastic recoil of the arteries forces the blood out into the arterioles. Therefore, the elastic properties of the arteries help to convert the pulsatile flow of blood from the heart into a more continuous flow through the rest of the circulation.

Many studies explored the importance of pulse in preserving tissue perfusion. In 1954, Burton showed that capillary flow ceased after arterial pressure decrease under critical closing pressure and that pulse prolonged the period of capillary opening (16). Several years later, in 1960, Teked**a** et al showed in an animal experiment that nonpulsatile flow caused a collapse of capillary structure, reduction in blood flow, and increase in capillary shunting, irrespective of the mean blood flow and arterial pressure (17). Prior et al (18) suggested that pulse pressure profile at the capillary level, along with mean blood pressure and extracellular osmotic pressure, was the main factor responsible for the maintenance of fluid balance and exchange of nutrients at the cellular level. Further study by Baba et al (19) confirmed the rapid decrease in erythrocyte velocity within the capillaries during nonpulsatile flow, with the reversal when pulsatile flow was resumed. Perfused capillary density was also reduced. They also suggested that a decrease in nitric oxide release in the microvessels due to nonpulsatile flow led to constriction of arterioles.

While these and many other studies of hemodynamics, metabolism, organ function, microcirculation, and histology show benefits derived from pulsatile perfusion, others do not. A possible explanation for these inconsistencies is that different investigators employ different forms of pulsatile perfusion, only some of which are effective. Studies do not adequately quantify the pulsatile components of flow, which makes it difficult to differentiate between ineffective forms of pulsatile flow and make comparison between studies (14). Additionally, in clinical studies other confounding factors impede pulse delivery, such as anesthesia, use of vasoactive agents, and temperature.

Pulsatility is usually described by arterial pulse pressure and pulsatility index. Pulse pressure (PP) is the difference between the maximum and minimum pressure, while pulsatility index (PI) is the difference between peak systolic and minimum diastolic blood flow velocity, divided by the mean velocity during the cardiac cycle. However, pulsatile flow depends on the energy gradient, rather than the pressure gradient, as shown by Undar et al (20). Shepard et al (21) first postulated in 1966 that additional energy delivered to the tissues with pulsatile flow was responsible for keeping the peripheral circulation open, as well as for extracellular fluid exchange. By mathematical modeling, they showed that at the same mean pressure pulsatile flow provided 2.4 times as much energy as nonpulsatile flow. They also proposed energy equivalent pressure (EEP) as the best tool to quantify pulsatile and nonpulsatile pressure-flow waveforms (22). EEP is the ratio between the area under the curve of power and the area under the curve of flow at the end of the cardiac cycle (23). Undar also proposed surplus hemodynamic energy (SHE), calculated as difference between EEP and mean arterial pressure (MAP), as a novel method for precise quantification of different levels of pulsatility and non-pulsatility and their meaningful comparison (24). SHE was used by Travis et al (25) to show differences between continuous and pulsatile support. At low support levels, pulsatile support restored SHE to within 2.5% of normal values, whereas continuous support decreased SHE by more than 93% of the normal baseline value. At high support levels, pulsatile support augmented SHE by 49% over normal values, whereas continuous support further decreased SHE by 97% of the normal values.

As pointed out by Soucy (26), studies comparing pulsatile-flow and continuous-flow support have presented conflicting findings, mostly due to variations in device operation, support duration, and the criteria used to quantify pulsatility. Kinetic measurements mentioned above can better quantify pulsatile energies, particularly with the growing trend of additional speed modulation of LVADs.

## Continuous flow

Continuous flow is defined as non-pulsatile and independent of the cyclic pressure gradient (systolic and diastolic) (22). Continuous-flow left ventricular devices pump blood from the left ventricle to the aorta throughout the cardiac cycle, with reduced or absent arterial pulse pressure. However, residual cardiac activity of the left ventricle and vascular tone of the peripheral vessels prevent the occurrence of pure “non-pulsatile” flow in vivo. Even with high level of continuous support, with aortic valve closed, contraction of the left ventricle additionally increases preload of the pump. Therefore, most of the patients on continuous-flow devices develop some degree of “pulsatility” (27), which allows measurement of systolic and diastolic blood pressures using modern cuff devices.

Because of clinical complications associated with continuous flow, efforts have been made to generate pulsatility with continuous-flow devices. Experiments from the 1990s showed that it was possible to generate pulsatile pressure similar to that of native physiology using a centrifugal LVAD (28). It seems that centrifugal pumps are able to generate pulsatility with fewer suction events when compared to axial-flow devices (29). One of the methods to produce an artificial pulse in continuous-flow devices is by modulating LVAD rotor speed. Bourque et al (30) sharply alternated the speed of the magnetically levitated rotor of the continuous-flow HeartMate III (Thoratec, Pleasanton, CA, USA) between 1500 rpm (artificial diastole) and 5500 rpm (artificial systole) at a rate of 60 bpm at a “systolic” interval of 30%, which produced pulse pressure in the range of 30 mm Hg. EEP did not exceed the mean arterial pressure. They concluded that very rapid speed changes can be used to simulate physiologic pulse pressure. Ising et al (31) used a computer model of the circulatory system simulating heart failure to study the effects of timing and synchronizing the continuous LVAD flow modulation with the native heart. By assessing over 150 different computer algorithms of varying pulse widths, beat frequencies, time shifts, and amplitudes, they found that synchronous speed modulation provided the greatest reduction in LV external work (LVEW), but that asynchronous modulation more dramatically increased EEP and SHE. These algorithms resulted in an increased arterial pressure pulsatility of up to 59 mm Hg, reduced left ventricular external work (LVEW) by 10%-75%, and increased myocardial perfusion by up to 44% from baseline heart failure condition. These studies show that modulation of continuous flow can provide pulsatility, but it still needs to be proven if it will be sufficient to prevent continuous-flow adverse effects, normalize vascular responses, and promote myocardial recovery.

## Left ventricular unloading and pulmonary artery pressure reduction

The optimal degree of LV unloading during VAD support has not yet been determined. The purpose of LVAD is to entirely off-load the heart while restoring systemic blood pressure. Complete resting of the myocardium is supposed to be beneficial for myocardial recovery and should maximize myocardial perfusion. Both animal models and clinical studies have observed some differences in left ventricular unloading patterns between continuous and pulsatile devices.

Koenig et al (32) showed in mock circulation that continuous assist reduced filling pressures (mean left atrial and LV end diastolic pressure) by 50% more than pulsatile devices with downward shift of pressure-volume (P-V) loop. However, these benefits were at the expense of a higher mean distal aortic pressure and lower diastolic to systolic coronary artery flow ratios. Such findings suggest the potential for differences in endocardial perfusion between assist techniques. Bartoli et al (33) developed a chronic ischemic heart failure bovine model using coronary microembolization. They analyzed the acute effects of different flow modalities on the left ventricle and found that continuous flow VAD support provided greater LV unloading than pulsatile support, characterized by lower LV end-diastolic and end-systolic volumes, reduced LV end-diastolic and end-systolic pressures, and increased diastolic aortic pressure. However, increased continuous unloading led to the collapse of pressure-volume relationships and the aortic valve remained closed. In contrast, as pulsatile unloading increased, a comparable decrease in left ventricular volumes was noted with preservation of a normal range of left ventricular pressures. Continuous unloading deranged the physiologic profile of myocardial and vascular hemodynamic energy utilization, whereas pulsatile unloading preserved more normal physiologic values.

Garcia et al (34) compared LV unloading between pulsatile device (HeartMate XVE, Thoratec) and continuous-flow device (HeartMate II, Thoratec) in 25 patients. After 1 month of support, substantial left ventricular unloading and hemodynamic improvement was achieved using both devices with no statistically significant difference between them. Kato et al (35) analyzed the effects of ventricular decompression on myocardial structure and function by continuous-flow and pulsatile-flow LVADs in 61 patients who underwent LVAD implantation as bridge-to-transplant. They found that mechanical unloading of the failing myocardium was more effective using pulsatile devices, as indicated by echocardiographic parameters of systolic and diastolic LV function as well as dynamics of BNP and ECM markers. Klotz et al (36) demonstrated in a series of 31 patients that pulsatile-flow pumps (Novacor [Swanley, UK] LVAS and TCI-HeartMate VE LVAS) provided greater LVAD outflow than continuous-flow pumps (MicroMed DeBakey VAD, MicroMed Houston, TX, USA), as well as greater reduction in echocardiographic end-systolic measurements (dimension and volume). Continuous-flow LVADs generated only partial LV volume unloading, whereas pulsatile LVADs produced complete volume unloading. However, the reduction of mean pulmonary pressure and pulmonary capillary wedge pressure was the same in both device types.

Several studies have proved that adequate left ventricular decompression can reverse even significant pulmonary hypertension. In the study of John et al (37), 50 patients were bridged to transplant with continuous-flow LVAD (HeartMate II). After LVAD placement both mean pulmonary artery pressures and mean pulmonary vascular resistance decreased significantly from baseline values. Posttransplant pulmonary hemodynamics also remained within normal limits, even in patients with previously severe pulmonary hypertension. Ozturk et al (38) compared the efficacy of continuous and pulsatile-flow pumps to reduce pulmonary hypertension. Fifteen of 27 patients had continuous-flow pump (HeartWare, Framingham, MA, USA) and 12 had pulsatile pump (Berlin Heart EXCOR, Berlin, Germany). A significantly greater decrease in systolic PAP was observed in patients with continuous-flow blood pumps, but there was no statistically significant difference between the groups in change in TAPSE.

To conclude, it appears that pulsatile and continuous-flow devices provide similar hemodynamic pressures, flows, and ventricular unloading. Differences observed in some studies could mostly be attributed to the higher level of pulsatile device support due to purposely lower operating speed of continuous-flow devices (to prevent suction events and permit intermittent opening of the aortic valve).

## Vascular reactivity and histology

Pulsatility with its intrinsic properties of cyclic strain and shear stress leads to complex signaling process in the vascular endothelium, often termed mechanotransduction (39). Endothelial cells in vivo act as a signal transduction interface (“biosensor”) for hemodynamic forces in the acute regulation of artery tone and chronic structural remodeling of arteries, perhaps even in the pathology of atherosclerosis. Endothelial cells seem to respond differently to continuous than to oscillatory shear stress (40). Pulsatility is a function of maximum flow rate (41), which implicates that pulsatile flow generates higher wall shear stress than equal continuous flow. Systemic vascular resistance could be reduced by increasing either pulse pressure or pulse rate (42). Both of these factors significantly and independently stimulate vasodilation in vivo by increasing endothelial production of nitric oxide (43). This explains progressive systemic arterial vasoconstriction during continuous-flow support observed in several studies (44,45).

Habazettl et al (46) used intravital microscopic technique to directly observe diameters and blood ﬂow velocity in sublingual microvessels in a patient scheduled for LVAD implantation. They found that 60% increase in pump speed directly translated into a similar increase (76%) in mean arteriolar blood flow velocity, whereas mean arterial pressure increased by only 21%, indicating that microvascular perfusion *in vivo* largely depends on the performance of the pump and is not reflected directly by arterial pressure. They speculated that lack of flow velocity oscillations may have profound long-term effects on shear stress-regulated arteriolar remodeling.

The impact of pulsatility on arterial wall histology has been assessed in several studies. Cyclic mechanical strain leads in vitro to proliferation of vascular smooth muscle myocytes by producing autocrine platelet-derived growth factors (15). Nishimura et al (47) showed in their animal experiment that non-pulsatile flow reduced wall thickness of the aorta and volume ratio of vascular smooth muscle myocytes, and increased the proportion of vascular smooth muscle cells with low activity and low contractility. They also reported that the systemic vascular resistance response to norepinephrine infusion decreased markedly in prolonged nonpulsatile circulation, although the plasma norepinephrine level was not changed (48). In the study by Kihara et al (49), more pronounced vascular smooth muscle cell hypertrophy in renal cortex arteries was related to lower speed of continuous-flow LVADs implanted in calves. However, Potapov et al could not identify any relevant difference in arterial wall characteristics between groups of patients with pulsatile-flow (9 patients) and continuous-flow devices (16 patients) in a histological study of tissue obtained from patients supported for more than 180 days (50). They concluded that long-term mechanical circulatory support with continuous-flow devices did not adversely influence arterial wall properties of the end-organ vasculature.

## End-organ function

The role of pulsatility in microcirculation is uncertain, due to the reduced pulse pressure at the capillary level. Also, after decades of clinical experience with both pulsatile and continuous-flow support, we know that both flow modalities reverse end-organ dysfunction in terminal stage HF patients and provide good end-organ function even during extended support. However, potential adverse effects of continuous flow on end-organ perfusion were more investigated in both animal and clinical studies.

In several studies Sezai et al (51-53) have compared the recovery of microcirculation in pigs using biventricular support after induced myocardial infarction. These studies showed that continuous circulation was not as effective as pulsatile in recovering microcirculation of the kidney, liver, stomach, and skin. However, there were no significant differences between the groups in white and gray matter regional blood flow and carotid arterial flow. Saito et al (54) compared end-organ function between pulseless and control sheep using Terumo magnetically suspended centrifugal pump. In both groups all measures of end-organ function remained within normal range. They also found no histologic differences between the organs of animals from pulsatile and nonpulsatile group, except for thinning of the medial layer of the ascending aorta in nonpulsatile group. The renin-angiotensin system was up-regulated in the nonpulsatile group, without a significant rise in blood pressure. Contrary to this, Golding (55) could not find a significant change of plasma renin activity in up to 3 months of nonpulsatile support.

Letsou et al (56) reported that 10 patients supported for up to six months with continuous axial-flow device (Jarvik 2000, Jarvik Heart, New York, NY, USA) had both renal and hepatic function preserved. They concluded that concerns about impaired end-organ function due to the dampened pulsatility of continuous axial-flow devices appeared to be unjustified. Radovancevic et al (57) reported long-term effects of different flow modalities in patients supported for more than 6 months. In both continuous-flow (12 patients, Jarvik 2000 or Thoratec HeartMate II) and pulsatile group (58 patients, Thoratec HeartMate I), albumin, blood urea nitrogen, creatinine, creatinine clearance, total bilirubin, and transminase levels either improved or stayed within the normal range at 6, 9, 12, and 15 months after LVAD implantation. Kamdar et al (58) compared renal, hepatic, and hematologic function in three groups of patients supported up to three months by either centrifugal-flow, axial-flow, or pulsatile LVAD. In all groups, both renal and hepatic functions were normalized, with no differences in end-organ function between the continuous and pulsatile devices.

Both experimental and clinical evidence suggests that pulsatility is not an essential requirement for a LVAD device. With continuous-flow pumps, end-organ function is well preserved although pulsatility may accelerate recovery from cardiogenic shock.

## Aortic valve changes

As a consequence of long-term LVAD use two seemingly different changes of the aortic valve have been described: aortic insufficiency and aortic fusion. Aortic insufficiency (AI) occurs quite commonly in patients supported with LVAD. In a recent meta-analysis of 7 observational studies (657 patients) by Deo et al (59), incidence of AI was 25%, with 4% increase per month of support. Sixty-five percent of patients underwent implantation with a continuous-flow device. More likely to develop AI were destination therapy patients (odds ratio 5.3), patients with continuous-flow pumps (hazard ratio 2.2), and patients with closed aortic valve (odds ratio 4.7), and survival was comparable in both cohorts. Pak et al (60) previously also confirmed that patients supported by HeartMate II continuous-flow pump were two times more likely to develop aortic insufficiency than patients supported by HeartMate XWE pulsatile pump (14.3% vs 6.0%). Another study confirmed that aortic insufficiency was more common in cases when the aortic valve did not open (61). This study compared AI frequency of different continuous-flow LVADs with that of a pulsatile LVAD and again confirmed that continuous flow and aortic valve opening were risk factors for AI.

Due to the diminished pulse pressure in continuous pumps, outflow produces constantly elevated transvalvular pressure gradient and increasingly stresses the walls of the left ventricle, aortic valve, and aorta. It is easy to grasp that alterations and redistributions of stress can lead to soft tissue remodeling (62). A biomechanical study of aortic valve leaflets during long-term support (63) indicated that average strain was increased due to augmented minimum systolic strain. Elevated pressure gradient and strain may lead to AI, reduced valve opening, and aortic root or valve leaflet remodeling. Researchers suggested that structural remodeling may also be caused by nutrient deprivation as a result of compression due to strain. Cowger et al (64) compared acquired AI after implantation of the pulsatile (HeartMate XVE, 25 patients) and continuous-flow pump (HeartMate II, 53 patients). The continuous-flow pump patients had an increased incidence and severity of AI, but in no patient was AI severe enough to require intervention.

Aortic valve fusion is defined as the deposition of loose, fibrous tissue joining the commissures of two coapting leaflets, which results in a compromised and incomplete opening of the valve during systole. It can be caused by previously mentioned remodeling of the aortic root and valve leaflets. A retrospective evaluation of samples from HeartMate II BTT patients found that 8 of 9 patients had evidence of commissural fusion of the aortic valve leaflets (65). Pathogenesis of aortic valve fusion was also shown by Rose et al in a series of explanted hearts after pulsatile LVAD use (66). In this study pumping algorithm kept the aortic valve permanently closed, leading to stasis on the ventricular aspect of the aortic valve and thrombus formation and organization, which led to aortic stenosis of variable severity. Martina et al (67) found fusion of single or multiple commissures in 11 of 19 (58%) hearts from patients supported by either HeartMate II or HeartWare continuous-flow LVADs. Commissural fusion was associated with continuous aortic valve closure during support and had induced aortic valve insufficiency in all patients with fusion.

Data suggest that the critical difference between continuous and pulsatile-flow support with respect to AI and aortic valve fusion is the occurrence of aortic valve opening, which is protective for both disorders. Minimal opening of the native aortic valve interrupts commissural fusion and protects against the development of AI by temporarily interrupting the stress of systolic pressure on the closed commissures (68). This protective effect was confirmed by the lack of clinically important AI on echocardiography in any of the patients supported by the pneumatic pumps.

## Bleeding events

Bleeding complications remain the most serious adverse events in the current era of mechanical circulatory support therapy (12,69,70). Non-surgical bleeding is one of the most common adverse events even in the early postoperative period. The most commonly reported sources of bleeding are epistaxis, gastrointestinal (GI) tract bleeding, bleeding of the mediastinum and thorax, and intracranial hemorrhage (71). The incidence of postoperative bleeding has been significantly reduced by using continuous-flow pumps (12), as well as the incidence of surgical reexploration for bleeding (still about 20% of patients require reopening) (72,73). Although being the most feared complication, hemorrhagic infarction fortunately remains infrequent adverse event, with published rates ranging from 0.01 to 0.08 events per patient-year (70,74,75). Since the first report of 3 cases of chronic GI bleeding during continuous-flow LVAD support in 2005 (76), it remains one of the most debated topics in the MCS community.

There are multiple underlying mechanisms potentially causing disorder of hemostasis, including loss of platelet number and impaired aggregation, acquired von Willebrand syndrome, activation of the fibrinolytic system, angiodysplasia, and arteriovenous malformations in GI system. Hemostasis is also adversely influenced by other factors, such as anticoagulation and/or antiplatelet therapies, as well as preexisting hepatic dysfunction.

Patients with continuous-flow LVADs have a higher rate of gastrointestinal bleeding events than pulsatile LVAD recipients. In the study by Crow et al (77), the event rates were 0.63 gastrointestinal (GI) bleeding events per patient-year for nonpulsatile devices and 0.068 events per patient-year for pulsatile devices. This 10-fold difference persisted also for bleeding occurring 31 days after implantation or longer (0.465 events per patient-year vs 0.047 events per patient-year). Higher GI bleeding incidence after implantation of continuous-flow devices was noted by Stern et al (78), who found that 40% of HeartMate II recipients had suffered from at least one episode of GI bleeding. In a recently published series by Demirozu et al (79), 53 gastrointestinal bleeding episodes were recorded in 32 patients (of 172 implanted with HeartMate II), providing important information on the location of bleeding: 16 patients with upper, 15 with lower, and 1 with both upper and lower GI bleeding. Arteriovenous malformation was identified as the source in 10 of 32 patients (31%). Muthiah et al (80) published a retrospective analysis of 66 patients implanted with centrifugal continuous-flow LVADs (Ventrassist [Ventracor, Sydney, Australia] and HeartWare). Bleeding GI angiodysplasia was demonstrated in 5 out of the 12 (41.6%) patients who underwent endoscopy from the cohort of supported patients (7.6%). The incidence of bleeding angiodysplasia was higher than the age-standardized rate of angiodysplasia from literature (0.8%). They suggested that it may be appropriate to screen for angiodysplasia particularly in older patients prior to support by centrifugal-flow LVADs.

Acquired von Willebrand syndrome is observed in patients supported by continuous-flow devices. Type 2A von Willebrand syndrome is characterized by the loss of the largest von Willebrand factor multimers, which are most effective in platelet-mediated hemostasis (81). Similar to acquired von Willebrand syndrome seen in patients with aortic stenosis (Heyde’s syndrome) (82), the shear stress of the continuous-flow LVAD may cause proteolysis of the high molecular weight (HMW) multimers (83). Giesen et al (84) were first to evaluate hemostasis parameters in HeartMate II, Thoratec BiVAD, and heart transplant recipients within 30 days after the surgical procedure. Large vWF multimers were missing in all of 10 tested VAD patients, whereas 5 of 6 tested HTX recipients displayed normal multimer pattern. Even though there are elementary mechanical differences between two analyzed VAD systems, both systems caused an acquired von Willebrand syndrome. Meyer et al (85) established von Willebrand syndrome type 2 in all 26 patients receiving continuous-flow LVAD (HeartMate II; Thoratec). Bleeding events occurred with an incidence of 0.17 per patient-year, ranging from epistaxis to life-threatening GI bleeding (source distal of the duodenum in the small bowel). Restoration of a normal vWF monomer pattern was found in all 12 transplanted or recovered patients. Meyer’s findings may support the belief that induced hypocoagulability and von Willebrand syndrome are an important characteristic of continuous-flow pumps use, thus reducing the overall need for anticoagulation. The loss of large vWF multimers was also noted in rotary blood pumps other than HeartMate II (86). vWF profiles and vWF high molecular weight multimers were measured pre- and post-LVAD placement in 11 nonpulsatile (HeartMate II, Thoratec) and 3 pulsatile (HeartMate XVE, Thoratec) recipients in a study by Crow et al (87). They concluded that continuous pump recipients developed HMW multimer loss and impaired vWF platelet-binding ability after LVAD placement, unlike a small series of pulsatile device recipients. Altered vWF multimers have been documented after the change from pulsatile VAD (HeartMate XVE, Thoratec) to continuous-flow pump (HeartMate II, Thoratec) (88).

In brief, continuous flow is the most probable culprit of acquired von Willebrand syndrome development, which could uncover previous subclinical arteriovenous malformations. Additional factors could include distention of submucosal venous plexus from diminished pulsatility, increased intraluminal pressure, as well as potential effects of vWF on angiogenesis (89).

## Thrombosis

Potential thrombosis remains one of the problems of mechanical circulatory support therapy. Thrombosis can take the form of thromboembolic cerebrovascular infarction, pump thrombosis, and aortic root thrombosis.

Up to 16% of patients in REMATCH trial (1) had cerebrovascular accident, with a very high rate of 0.19 events per patient-year (90). Unlike pulsatile-flow devices, continuous-flow devices have been associated with a lower rate of thromboembolic events. In a report of John et al (91), only one of 45 patients supported with HeartMate II LVAD experienced a thromboembolic event. In other studies, rate of non-hemorrhagic cerebrovascular infarction was between 0.05 and 0.09 events per patient-year (74,92,93).

Pump thrombosis can occur both early and late after LVAD implantation and in continuous-flow devices it occurs in the range of 0.01 to 0.05 events per patient-year (74,75,93). Both verification and management of this dreadful complication are complex. Usually it presents with pump power increase, hemolysis, or signs of cardiac decompensation; echocardiography or right heart catheterization can help in the diagnosis. Thrombolytic therapy and pump exchange have been proposed as treatment options.

Although quite uncommon, aortic root thrombosis can occur during continuous-flow LVAD support. Several case reports described thrombosis of the aortic root and ascending aorta (94-96), which can be further complicated by myocardial infarction due to the left main coronary artery occlusion (97). The rate of thromboembolism during long-term outpatient support with the continuous-flow devices is low but seems to be offset by a higher rate of hemorrhagic events.

## Conclusion

After more than a decade of clinical experience with continuous-flow support in HF patients, it is well proven that continuous flow provides significantly better outcomes, higher quality of life, and lower adverse event rates when compared to previous generations of pulsatile-flow devices. Numerous animal and clinical studies confirm that there are no major differences between continuous and pulsatile-flow devices in terms of left ventricular unloading and end-organ function. However, mounting evidence shows that unanticipated consequences of continuous-flow support – namely acquired aortic valve insufficiency and acquired von Willebrand syndrome – can cause a real clinical problem. Some of these difficulties should be resolved by incorporating pulsatility into current and emerging continuous-flow devices.
